# A commentary on ‘The safety and efficacy between remimazolam and propofol in intravenous anaesthesia of endoscopy operation: a systematic review and meta-analysis’

**DOI:** 10.1097/JS9.0000000000000710

**Published:** 2023-10-09

**Authors:** Junhong Chen, Hao Zhou, Kai Liu

**Affiliations:** Department of Hepatobiliary and Pancreatic Surgery II, General Surgery Center, The First Hospital of Jilin University, Changchun, People’s Republic of China

*Dear Editor*,

As a new ultra-short-acting benzodiazepine anesthetic, remimazolam is exerting an important impact on endoscopic sedation^1^. Therefore, we read with great interest the recently published meta-analysis in the *International Journal of Surgery* that compared the safety and efficacy of remimazolam with that of the traditional sedative propofol^2^. While we commend Zhao *et al*. for this timely work, which provides a high level of evidence for remimazolam use in endoscopic procedures, we would like to point out several issues regarding the statistical framework used in this meta-analysis that may compromise the reliability of their conclusions.

Firstly, the unit-of-analysis problem needs to be considered in this meta-analysis^3^. How to include studies comparing multiple experimental groups for meta-analysis needs to be treated with caution. For example, for a study with ‘dose1 versus control’ and ‘dose2 versus control’ (two comparisons share the same control group), if several comparisons are simply entered into a meta-analysis, participants in the shared group will be double-counting, thus leading to a serious unit-of-analysis problem^3^. Specifically, three of the seven studies included in the present meta-analysis had multiple experimental groups based on the dose of remimazolam, but they all had only one control group, the constant-dose propofol group^2^. The authors simply included several comparisons from the same study together in the meta-analysis, which meant that the propofol group was double-counted among the participants. The double-counting of participants spuriously increased precision and resulted in an elevated risk of false positive conclusions. Unfortunately, 75% of adverse events (3/4: injection pain, hypotension, respiratory depression) and 100% of secondary outcomes (3/3: operation completion rate, time for fully alert, time for discharge) meta-analyses in the study by Zhao *et al*. were affected by this bias. Take the hypotension outcome, for example the included Dai-2020 and Tan-2022 reported three and two remimazolam groups with different doses, respectively, while both had only one propofol group. Therefore, when pooling effect sizes, participants in the propofol groups of the two studies were repeatedly included three and two times, respectively. Ultimately, the effect sizes of these two studies accounted for 63.8% of the meta-analysis weights, while the number of their participants accounted for only 29.4% of the five studies included^2^. In fact, the Cochrane Handbook has provided several ways to address such multi-arm studies^3^:

1. Combine multiple relevant experimental groups to create a single pairwise comparison. In most situations, this approach is recommended.
2. Choose one pair of interventions while excluding others.
3. Split the ‘shared’ group into several groups with smaller sample sizes and include all reasonably independent comparisons.
4. Perform network meta-analyses.

Secondly, the methodology for assessing the risk of publication bias was incorrectly applied. Publication bias is a common problem in clinical research, which can lead to overestimation of benefits and underestimation of harms^4^. Zhao *et al*. used the funnel plot to examine publication bias and claimed that its risk was low for all endpoints except the time to discharge^2^. However, the funnel plot seems not to be applicable to the present meta-analysis. As a graphical tool relying on visual inspection, the funnel plot can only be used if the number of included studies is ten or more, otherwise it does not have sufficient testing power^4^. However, the authors included only seven studies in total, and even among the seven endpoints using the funnel plot, most (4/7) included fewer than five studies. Thus, the funnel plot may not provide reliable estimates of publication bias for the present meta-analysis. For example, the funnel plots for hypotension and bradycardia were not as symmetrical as the authors stated, and perhaps the small-study effects should be suspected^2^.

Thirdly, the authors set *I* ^2^<50% and *P*=0.1 as the criteria for no substantial heterogeneity and used the fixed-effects model^2^. However, *P*=0.1 is not reasonable, and here perhaps it should be *P*>0.1. In the subsequent meta-analysis of bradycardia, the fixed-effects model was incorrectly used in cases of heterogeneity as high as *I* ^2^=59% and *P*=0.09. The opposite error occurred in the subgroup analyses of time for discharge, with *I* ^2^<50% and *P*>0.1 for both subgroups, but incorrectly using the random-effects model instead of fixed-effects.

Fourthly, the screening process seems flawed, as the sum of included and excluded studies doesn’t equal the initial records.

In conclusion, we thank Zhao *et al*. for this excellent work. Meta-analyses can provide the highest level of evidence for evidence-based medicine and guide clinical practice, but appropriate methodology is required. We hope to raise the above issues so that readers of the *IJS* will have a more accurate understanding of the results of the comparison between remimazolam and propofol.

## Ethical approval

Not applicable.

## Consent

Not applicable.

## Source of funding

None.

## Author contribution

K.L. and J.C.: conceived this manuscript; J.C. and H.Z.: wrote the manuscript.

## Conflicts of interest disclosure

There are no conflicts of interest.

## Research registration unique identifying number (UIN)

1. Name of the registry: not applicable.
2. Unique identifying number or registration ID: not applicable.
3. Hyperlink to your specific registration (must be publicly accessible and will be checked): not applicable.

## Guarantor

Prof Kai Liu, MD.

## Data availability statement

Not applicable.

## Provenance and peer review

Not commissioned.
